# The Fate of Maleic Hydrazide on Tobacco during Smoking

**DOI:** 10.1100/2012/451471

**Published:** 2012-10-23

**Authors:** Hongfei Zhang, Gangling Tang, Nan Liu, Zhaoyang Bian, Qingyuan Hu

**Affiliations:** Chemical Analysis Laboratory, China National Tobacco Quality Supervision & Test Center, Zhengzhou 450001, China

## Abstract

Tobacco mainstream smoke (MSS) and sidestream smoke (SSS), butts, and ashes from commercial cigarettes and maleic hydrazide (MH) spiked cigarettes were analyzed for their MH contents. The MH transfer rates obtained for MSS ranged from 1.4% to 3.7%, for SSS ranged from 0.2% to 0.9%, and for butts ranged from 1.1% to 1.9%. And as expected, MH is absent in ashes. The transfer rate of MH into mainstream smoke is the top one during in transfer rate into main-stream, side-stream, ashes, and butts, and higher MH levels lead to more MH in smoke. Further, analysis of total MH in butts and ashes along with that in MSS and SSS indicates that much MH is destructed during the smoking process.

## 1. Introduction

Maleic hydrazide (MH; 1,2-dihydro-3,6-pyridazinedione), a systemic plant growth regulator, is used by tobacco farmers throughout the world to inhibit growth of suckers on tobacco plants [1]. It is usually applied to the upper half or third of the tobacco plant within 24 h after topping. Subsequently, due to absorption and translocation, MH is found throughout the entire plant. However, this compound is suspected to be a carcinogen [2] and is classified as group C by the International Agency for Research on Cancer (IARC). The total level of MH in the processed leaf and in the tobacco from cigarettes ranges from a few *μ*g/cig up to about 100 *μ*g/cig. The German government and the CORESTA have set a tolerance level of 80 ppm of this substance in cigarette tobacco [3]. However, as far as the smoker is concerned, the most important question is not how much MH is in tobacco but how much of MH and its degradation products is present in cigarette main-stream smoke? Our investigations are, in part, an answer to that question. 

Pyrolysis at 750°C of pure MH, performed in the present study, indicates that at elevated temperatures some of MH is transferred unmodified into the pyrolyzate. The transfer of MH from tobacco to cigarette smoke is therefore likely. Several previous studies report MH transfer into smoke. Liu and Hoffmann [4] were the first to report that the transfer rate of MH depends on the concentration of MH in tobacco. The transfer rate indicated by Liu and Hoffmann [4] ranged from 4% to 10%. Haeberer and Chortky [5] found a transfer rate of only 0.2%. A report by the US Department of Agriculture [6] indicated a transfer of 2-3% of MH to mainstream smoke (MSS), while Chopra et al. [7] showed values between 2.5% and 2.8%. Additionally, Liu et al. [8] indicated that MH is present in smoke even if the tobacco was not treated with MH.

The disagreement between the reported results of MH transfer rate indicated a need for further studies. Our investigation was done to clarify the reasons for differences among previously published results. It started with the pyrolysis of pure MH to establish the capability of MH to be transfered unmodified into pyrolyzates. Further, MHs were applied to cigarettes by injecting their solutions into the cigarettes. Then, we have analyzed cigarette tobacco, cigarette mainstream and side-stream smokes (SSS), cigarette butts, and cigarette ashes for their MH contents and have calculated the transfer of MH into mainstream and side-stream smokes.

## 2. Experimental

### 2.1. Reagents and Standards

The materials used for the experimental part of this study are as follows: MH from Chem Services Inc. (West Chester, PA, USA), and BSTFA from Regis Technologies Inc. (Morton Grove, IL, USA), dimethylformamide (DMF) from Burdick and Jackson (Muskegon, MI, USA), and tert-butylhydroquinone from Aldrich (Saint Louis, MO, USA). Cigarettes are smoked by a Borgwaldt KC 5 port sidestream smoking machine. All cigarettes were stored at 0°C until required for use. The 3R4F cigarettes were purchased in 2010 (University of Kentucky), and the commercial cigarettes, all different brands, were purchased in 2011 from local grocery stores.

### 2.2. Pyrolysis of MH

The pyrolysis of pure MH was done using an on-line pyrolysis-gas chromatography-mass spectrometry (Py-GC-MS) system. The on-line system use a CDS 5000 instrument and an Agilent 6890-5973 GC-MS system. The sample was pyrolyzed in the Pyroprobe at 750°C under helium for 10 sec, and the pyrolyzates were transferred to the GC-MS system by helium for analysis. The GC was equipped with a 30 m long, 0.25 mm i.d., and 0.5 *μ*m film thickness DB-Wax capillary column which has PEG-20 M functional groups. The oven was programmed at an initial temperature of 50°C for 2 min, a heating rate of 5°C/min to 200°C, a heating rate of 8°C/min to 280°C, and a final time of 15 min. The injection is done at 275°C. The quantitation was done using the area of the MH peak in the select ion chromatogram.

### 2.3. Sample Preparation

Some cigarettes were spiked with different levels of MH. For this purpose, a 10, 20 or 50 *μ*L solution of MH was injected, and the spiked cigarettes were equilibrated for 48 h before smoking. The concentration of MH in methanol was prepared to yield the desired amount of MH in the cigarette. For MH analyses in mainstream and sidestream smokes, 6 cigarettes per analyses were kept in a humidifying chamber for 24 h at 60% humidity. These cigarettes were then smoked onto a small Cambridge pad, under Federal Trade Commission (FTC) conditions using a Borgwaldt KC 5 port sidestream smoking machine. The main-stream and side-stream smokes were collected at the same time. Ashes and butts from these smoked 6 cigarettes were also collected and pooled together (separately). Each of MSS, SSS, butts, and ashes parts was then extracted with 50 mL methanol on a mechanical shaker for 20 min. The solution is transferred into a test tube, heated at 65°C under a N_2_ stream, and methanol is evaporated to 1 mL. The 1 mL solution is cleaned up by a C18 cartridge and is eluted by 5 mL methanol. Then the elution solution is transferred into a test tube, heated at 65°C under a N_2_ stream, and evaporated to dryness. The residue is dissolved in 1 mL DMF containing 100 *μ*g/mL tert-butylhydroquinone (internal standard), followed by the addition of 0.5 mL N,O-bis(trimethylsilyl)trifluoroacetamide (BSTFA). The solution is heated at 75°C for 30 min and analyzed by GC-MS using conditions similar to those used to analyze the pyrolyzate. 

**Figure 1:**
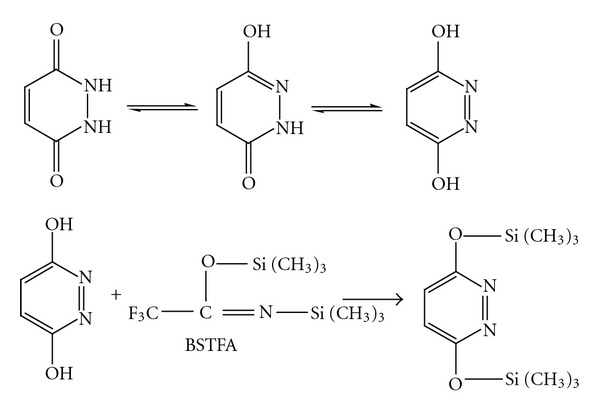
Tautomeric forms of maleic hydrazide and the reaction with N,O-bis(trimethylsilyl)trifluoroacetamide.

MH is present in several tautomeric forms, and in the presence of BSTFA, the enol form reacts as shown in Figure 1.

**Figure 2:**
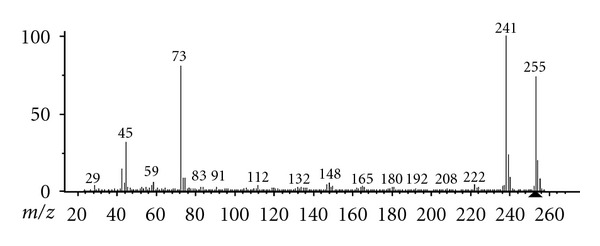
Mass spectrum of 3,6-bis[(trimethylsilyl)oxy]pyridazine (MH-2 TMS).

The mass spectrum of silylated MH or 3,6-bis[(trimethylsilyl)oxy]pyridazine is similar to that of silylated 2,5-dihydroxypyrazine and is shown in Figure 2. Select ion mode quantization ion of silylated MH is 241 and 255 quantization ion of internal standard is 207, and 222.

**Figure 3:**
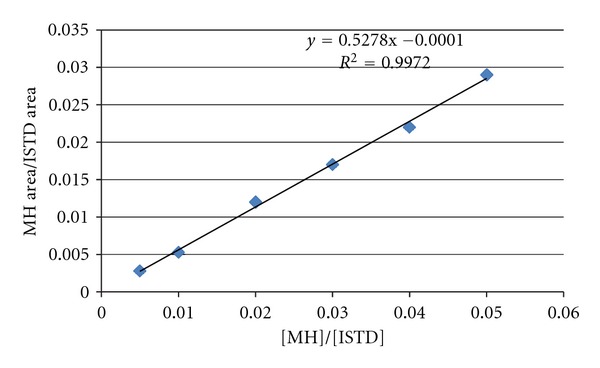
Calibration curve for the determination of MH in smoke.

The quantitation of MH in cigarette smoke is done following a calibration curve made using standards of pure MH with mainstream matrix. The standard solutions used to make the calibration curve are prepared starting with a stock solution of 1000 *μ*g/mL MH and 100 *μ*g/mL tert-butylhydroquinone in N,N-dimethylformamide (DMF). This solution is diluted by methanol to 100 *μ*g/mL MH with DMF containing the same concentration of internal standards, and various volumes from this solution were further diluted to generate each point. Three replicates of each concentration were prepared to generate the calibration curve. The calibration curve for MH in smoke is shown in Figure 3.

The matrix effect could affect silylation of smoke with BSTFA and yield low recovery. So matrix effect of main-stream, side-stream, ashes, and butts was comparative, and the recovery of each part was determinated.

The analysis of MH in tobacco is done following the next procedure. The analysis of total MH was performed by an external laboratory. The method used for this analysis is similar to that described in a literature report [9]. For the analysis, 5 g of tobacco was extracted by boiling under reflux in 100 mL of 4 N HCl for 120 min. The extract is allowed to cool and is filtered. A few mL of solution are passed through a C18 SPE column and a 4 mL sample is collected. A 10 *μ*L aliquot is injected in a high-performance liquid chromatography (HPLC) system equipped with a Varian Microsorb-MV 100-5 C18 column. The elution is done using isocratic conditions with 0.1 mol/L acetic acid in water (pH 4.8) at 0.7 mL/min. The optical density is measured at 313 nm.

## 3. Results and Discussions

### 3.1. Results of MH Pyrolysis

The decomposed part of MH consisted of some ammonia, CO_2_, butanoic acid, aminobutyric acid, 1H-pyrrole-2,5-dione, and so forth. The behavior of MH under pyrolysis conditions could be anticipated from its stability during melting point determinations. It was observed that MH does not melt but degrades at 295°C. This substantiates our findings of pyrolytic decomposition of MH in the burning cigarette. The result of MH pyrolysis indicates that a significant amount of MH passes unchanged into the pyrolyzate. The relatively high thermal stability of MH and its volatility allowed for part of the MH to volatilize and leave the pyrolyzer before decomposition. 

Our result indicates that beside MH itself, no specific toxicological concerns are raised by the presence of MH in cigarettes. But Patterson et al. [10] found benzo[a]pyrene in the pyrolyzate of MH when neat MH was prolyzed. This led to the possible implication that MH in cigarette tobacco could also contribute to benzo[a]pyrene in tobacco smokes. Chopra, however, on mathematical grounds, has questioned this implication [11]. Ninety-four percent of MH is known to degrade into CO_2_, CO, NH, HCN, and so forth [12]. This result is partly consistent with ours. And no one, so far, to our knowledge has experimentally implicated MH with PAHs. 

### 3.2. Silylation with BSTFA

Attempted application of our original method for MH in tobacco (silylation of MH and subsequent gas chromatography) to the analysis of MH in smoke was not immediately successful. Direct silylation of smoke with BSTFA yielded not good gas chromatogram. The analysis was complicated by interfering total particulate matter (TPM) constituents, not present in tobacco extracts. Removal of interfering substances was accomplished by C18 cartridge. Gas chromatography of the MH-BSTFA on DB-WAX showed that MH was successfully recovered from the C18 cartridge and derivatized quantitatively, in comparison with MH standards that were directly derivatized. Thus, the final procedure for MH analysis consisted of sample extraction, clean up of interferences by C18 cartridge, concentration and derivatization with the BSTFA reagent, and gas chromatography on DB-WAX.

### 3.3. Matrix Effect

**Figure 4:**
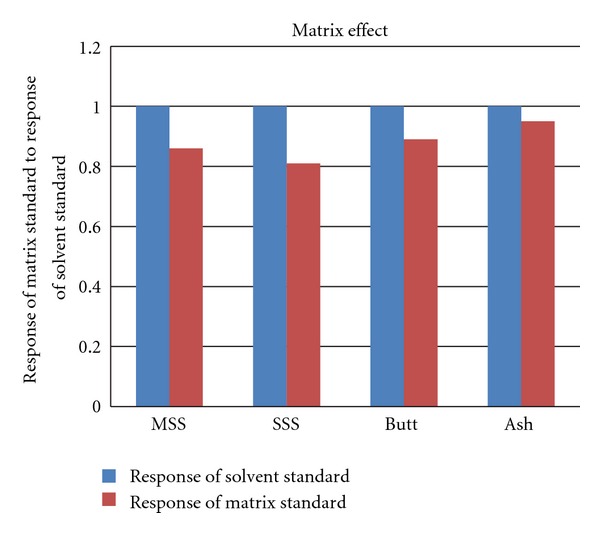
Matrix effects of main-stream, side-stream, ashes, and butts.

The matrix effect of main-stream, side-stream, ashes, and butts was comparative, as shown in Figure 4. We found that all of them have a matrix suppressive effect to MH; the strength order of matrix effect is side-stream, main-stream, butts, and ashes.

### 3.4. Recovery and RSD

Also the recovery and relative standard deviation (RSD) of each part were determined, as shown in Table 1. 3R4F cigarette (whose amounts of MH is close to zero) is used. We found that the average recovery of mainstream smoke is 83%, with RSD of 5.4%; the average recovery of sidestream smoke is 85%, with RSD of 4.9%; the average recovery of butts is 92%, with RSD of 5.8%; the average recovery of ashes is 94%, with RSD of 3.8%. The limit of detection is 0.12 *μ*g/cig, and the limit of quantitation is 0.39 *μ*g/cig.

### 3.5. Discussions of Transfer Rate

To obtain quantitative information on the transfer of MH in smoke, it was necessary to determine the levels of MH in both the tobacco and the smoke of each type of cigarette. Cigarettes with different levels of MH were used in this study. This included cigarettes with agronomically added MH in the tobacco and with spiked MH. The cigarette designs covered a range of possibilities, including 3R4F cigarette (whose amounts of MH are close to zero) and Parliament and Salem cigarettes. The levels of total MH and MH in smoke were analyzed as previously described. The results are given in Table 2. 

Our main-stream MH transfer rates are consistent with the few published results. Liu and Hoffmann [4] gave main-stream MH transfer rates of 3.94%. Chopra et al. [7] also gave main-stream MH transfer rates of 3.80%. This is in an excellent agreement with our main-stream MH transfer rate of 3.7%. However, Liu and Hoffmann [4] also report a transfer rate of 7% and 10.3%; our highest rate is 3.7% for a cigarette spiked 71.5 *μ*g/mL MH. Chopra et al. [7] have no explanation as to why Liu et al. [4] had such high transfer rates. But we found that Liu et al. [4] use water as extraction solvent in analyzing MH in tobacco; as we all know, water is not effective to extract bound MH in tobacco, so Liu got a low MH in tobacco and a high transfer rate of mainstream smoke.

Our main-stream MH transfer rates of 1.4% are also consistent with those of Davis et al. [13, 14] and Wood et al. [15]. It was of interest to determine whether or not any MH distills ahead of the burning cone of a cigarette. Apparently, sidestream smoke has a low transfer rate, which is due to the retention of moisture and particulate matter and so forth, by the butt segment during the process of smoking. Haeberer and Chortyk [16] report similar results. This indicated that MH does not distill ahead of the burning zone to be condensed and concentrated in the butt. Since distillation can now be ruled out, the disperse phase or aerosol particles must be responsible for transport of MH into smoke. This result is consistent with Chopra et al.'s [7].

Another interesting, and expected, finding is that the amount of MH is absent in ashes. The temperatures in the burning zone of the cigarette reach up to 900°C [17], and it would be surprising if MH could survive temperatures that high. 

## 4. Conclusions

This paper presents the fate of maleic hydrazide on tobacco during smoking. The transfer rate of MH into mainstream smoke is the top one during in transfer rate into main-stream, side-stream, ashes, and butts, and higher MH levels lead to more MH in smoke. The findings are in agreement with those of Liu and Hoffmann [4]. The most important consideration is not how much MH is in tobacco but how much MH is in main-stream smokes. According to our transfer rate, a cigarette containing 80 *μ*g MH would give 2.9 *μ*g MH in the mainstream smoke, 0.7 *μ*g MH in the sidestream smoke, and 1.4 *μ*g MH in the butt. 

## Figures and Tables

**Table 1:** Recoveries and RSDs of MH in MSS, SSS, butt, and ashes (*n* = 5).

Section	Added	Founded	Recovery	RSD	Average recovery	Average RSD
	*μ*g/cig	*μ*g/cig	(%)	%	(%)	(%)
MSS	1.43	1.09	76	6.3	83	5.4
	2.86	2.30	80	5.1		
	7.15	6.60	92	4.7		
	1.43	1.12	78	6.6		
SSS	2.86	2.35	82	4.0	85	4.9
	7.15	6.82	95	4.1		
	1.43	1.17	81	7.2		
Butt	2.86	2.62	92	6.2	92	5.8
	7.15	7.41	104	3.9		
	1.43	1.31	92	4.7		
Ashes	2.86	2.65	93	3.6	94	3.8
	7.15	7.03	98	3.3		

**Table 2:** The transfer rate of MH into MSS, SSS, butts, and ashes (%).

Kinds	MH in cigarette (*μ*g)	MSS	SSS	Butts	Ashes
MH-spiked samples					
3R4F + 14.3 *μ*g/cig	14.3	1.4	0.4	1.1	ND
3R4F + 28.6 *μ*g/cig	28.6	1.9	0.2	1.4	ND
3R4F + 71.5 *μ*g/cig	71.5	3.7	0.9	1.8	ND

Nonspiked samples					
Parliament	17.28	1.8	0.5	1.9	ND
Salem	8.97	1.8	0.3	1.2	ND
